# A comparative analysis of the karyotypes of three dolphins – *Tursiops truncatus* Montagu, 1821, *Tursiops australis* Charlton-Robb et al., 2011, and *Grampus griseus* Cuvier, 1812

**DOI:** 10.3897/compcytogen.v15.i1.60398

**Published:** 2021-02-11

**Authors:** Ross Brookwell, Kimberly Finlayson, Jason P. van de Merwe

**Affiliations:** 1 Cytogenetics Department, Sullivan Nicolaides Pathology, 24 Hurworth street, Bowen Hills, Queensland, 4006, Australia Cytogenetics Department, Sullivan Nicolaides Pathology Bowen Hills Australia; 2 Australian Rivers Institute, Griffith University Gold Coast, Edmund Rice drive, Southport, Queensland, 4215, Australia Griffith University Gold Coast Southport Australia

**Keywords:** Burrunan, chromosome, Common Bottlenose, G-band, Risso’s dolphin

## Abstract

The aim of this study is to produce G-banded karyotypes of three dolphin species, *Tursiops truncatus* Montagu, 1821, *Tursiops australis*Charlton-Robb et al., 2011, and *Grampus griseus* Cuvier, 1812, and to determine if any differences between the species can be observed. Monolayer skin cultures were established and processed for chromosome study by trypsin banding. The results indicate that the three species here investigated have the same diploid number (2n = 44) and very similar gross chromosome morphology, however G-banding allows distinction between each species. Chromosome 1 in *G. griseus* is significantly different from the other 2 species, and chromosome 2 in *T. australis* is subtly different from the other 2 species. This result is of potential significance in taxonomic studies, and can provide an unequivocal answer in the assessment of suspected hybrids between these species.

## Introduction

The family Delphinidae contains 37 recognized species, excluding *Tursiops australis*Charlton-Robb et al., 2011, whose status has not been conclusively resolved (Committee on Taxonomy 2020). The first karyotype of a dolphin, *Tursiops truncatus* Montagu,1821, was published by Walen and Madin (1965), and since then a total of at least seven species have been studied cytogenetically (Atlas of Mammalian chromosomes 2020). It has been concluded that the studied species have similar karyotypes, the majority of apparent variation being associated with differing accumulation of heterochromatic regions, as demonstrated in a study by G- and C-banding of the karyotypes of *Stenella clymene* Gray, 1850, *Lagenorhynchus albirostris* Gray, 1846, and *Phocoena phocoena* Linnaeus, 1758 (Arnason et al. 1980). There has, however, been no detailed comparative G-banding analysis of karyotypes within this family. This may in part be due to the use of differing banding techniques, the varying banding resolutions achieved, and use of differing karyogram templates, in addition to a general lack of availability of appropriate study material.

The three species of dolphin investigated here belong to the subfamily Delphininae, but it has been proposed that *Grampus griseus* Cuvier, 1812, should be attributed to the subfamily Globicephalinae, based on cytochrome *b* sequencing studies (LeDuc et al. 1999). The karyotype of *T. truncatus* has been published on several occasions, initially by Arnason (1974), and more recently with an ideogram by Bielec et al. (1997). The aim of this study is to describe the karyotype of *T. australis* and *G. griseus*, not yet described in the scientific literature, to enable use of the karyological characteristics of these species to identify putative hybrids between these species, and to help clarify the specific/sub-specific status of *T. australis*. The identification of hybrids is of interest because *T. truncatus* and *G. griseus* are the species most frequently noted as the origin of hybrids in captivity (Espada et al. 2019). There are conflicting views as to whether the recently described species *T. australis* (Charlton-Robb et al. 2011) should be categorized as such, or as a subspecies. It was considered on morphological grounds by Jedensjö et al. (2020) that *T. australis* falls within the species *T. truncatus*. A molecular study by Moura et al. (2020) provides evidence of a monophyletic origin of the genus *Tursiops* Gervais, 1855, but they conclude that their data indicate that *T. australis* is best considered as a subspecies within *T. aduncus* Ehrenberg, 1833 (refer to the phylogenetic network presented as Figure 2 in that paper, which clearly positions *T. australis* as a clade within *T. aduncus*). The molecular evidence for determining that *T. australis* is a separate species has been described as weak, and to include inappropriate analysis (WoRMS editorial board. 2020), and the morphological evidence has been criticized on the grounds that the sample size was small, interspecies comparison was limited and there was overlap occurring in all metric characters (Atlas of Mammalian chromosomes 2020).

## Material and methods

### Tissue source and cell establishment

The tissue samples available for this study were from a male and female common bottlenose dolphin (*T. truncatus*), a male and a female Burrunan dolphin (*T. australis*), and a female Risso’s dolphin (*G. griseus*). Skin samples from *T. truncatus* and *T. australis* were obtained from captive individuals at SeaWorld, Queensland, Australia during routine vaccinations. The tissue was taken from the tail using a biopsy punch. One female *T. truncatus* (CB01) was wild caught in 1994 and is approximately 33 years old. The other (CB02) was a male wild caught in 1985 and is approximately 43 years old. Both individuals of *T. australis* were born in captivity; one male aged 40 was transferred to Sea World in 1990 from Marineland, South Australia (BD01), and the other was a female aged 10 born at Sea World (BD04). A lung sample from a stranded *G. griseus* was provided by Dolphin Marine Conservation Park, Coffs Harbour, New South Wales, Australia (RD01). All tissue samples were immediately placed in DMEM media with 10% fetal bovine serum, 1% penicillin/streptomycin (10,000 U/mL stock) and 1% amphotericin B (250 μg/mL stock) and kept at 4 °C until processing.

Samples were washed several times with DMEM media (as described above) and cut into 1–3 mm pieces in fresh media. Tissue pieces were transferred to 25 cm^2^ flasks, arranged evenly on the lower surface of the flask. The flasks were incubated in an inverted position at 37 °C, 5% CO_2_ for 24 hours. Five mL of media was introduced, and then the flasks were returned to the incubator in an upright orientation. When cells reached ~70% confluence, tissue pieces were detached and removed. Cells were cryopreserved in liquid nitrogen at a concentration of 1 × 10^6^ cells/mL in DMEM media supplemented with 10% dimethyl sulfoxide, until ready to be used (Arsham et al. 2016).

### Species identification

The Qiagen DNeasy Blood and Tissue kit was used to isolate DNA from ~2×10^6^ cells, according to the manufacturer’s protocol for cultured cells. The resulting DNA was forwarded to the DNA Sequencing Facility at Griffith University, for confirmation of species. Around 660 bp of the mitochondrial COI gene was used for amplification by Platinum taq DNA polymerase (Invitrogen). The following primers were used – forward 5–3’ ATTCAACCAATCATAAAGATATTGG, reverse 5–3’ TAAACTTCTGGATGTCCAAAAAATCA (Hebert et al. 2004). ExoSap-IT (Applied Biosystems) was then used to clean the PCR amplicons, which were then bi-directionally sequenced. The Barcode of Life Database (v4, BOLD http://www.boldsystems.org/) was then used as a reference to classify the resulting sequences.

### Karyotyping

A flask of cells for each dolphin at various passages (CB01: P7; CB02: P6; BD01: P6; BD04: P6; RD01: P6) was forwarded to the cytogenetics laboratory at Sullivan Nicolaides Pathology. Here, the cells were either incubated overnight at 37 °C prior to initiation of harvest, or sub-cultured into 25 cm^2^ flasks in Amniomax II medium (Gibco), then incubated at 37 °C until ready for harvest. The cells were harvested when approximately 80% confluent. This was initiated by adding colchicine (100 µg/mL,Sigma) for 2 hours, suspending the cells in the medium with trypsin (Trypsin/EDTA 1×, Sigma), and swelling the cells by treatment with hypotonic solution (0.075 M potassium chloride) at 37 °C for 10 minutes. A 10% prefix solution of 3% acetic acid was then added before methanol/glacial acetic acid (3:1) fixation. The resulting cell suspension was used to prepare slides by dropping via a glass pipette onto clean dry slides (Arsham et al. 2016). After overnight incubation at 60 °C, the slides were G-banded using a modification of the method of Seabright (1971). Wright’s/Giemsa stain (Kinetik) was used to stain the slides.

A Metafer slide scanner (Metasystems) was used to select cells for processing, and the Ikaros karyotyping system (Metasystems) was used to produce karyograms.

The template employed for chromosome grouping is consistent with that used by Bielec et al. (1997), and their chromosome assignments have been followed as far as possible, given the difficulty sometimes caused by differences in appearance between replication banded and trypsin banded chromosomes. The chromosomes are arranged according to position of the centromere, pairs 1–2 are subtelocentric, pairs 3–11 submetacentric, 12–17 metacentric and 18–21 acrocentric, noting that some chromosomes could be categorized within different groups, but the template has been followed.

## Results

### Species identification

Species identification confirmed both CB01 and CB02 to be *T. truncatus* with a 99.27% and 99.85% match of COI gene sequence, respectively. BD01 and BD04 were confirmed to be *T. australis* with a 99.71% COI gene sequence match for both individuals. RD01 was confirmed to be *G. griseus* with a 99.85% match of COI gene sequence.

### Karyotype

**Figure 1.**
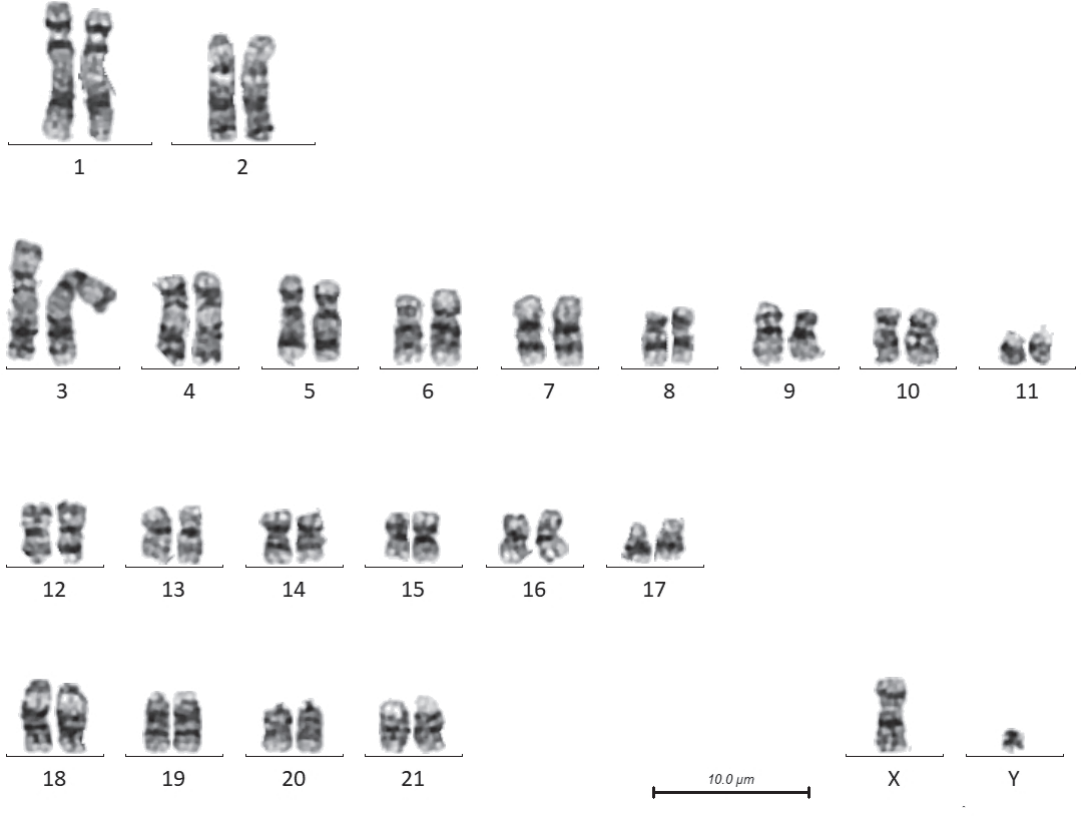
G-banded karyotype of male *T. truncatus* (CB02). Note the size polymorphism in the distal short arm of chromosome 6.

**Figure 2.**
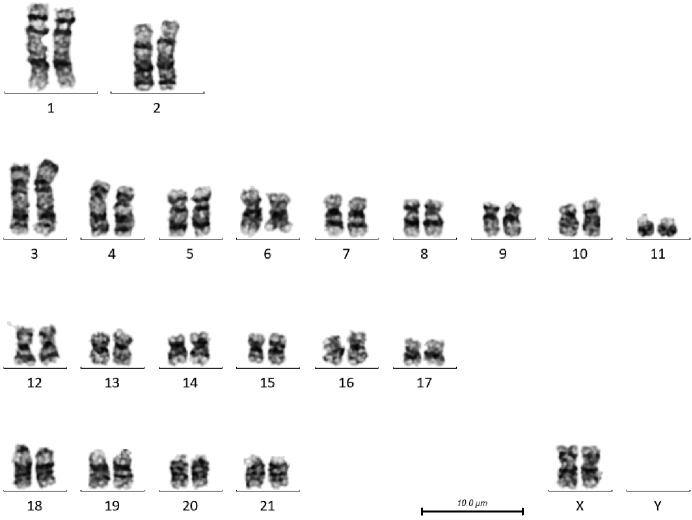
G-banded karyotype of female *T. truncatus* (CB01). Note the size polymorphism in the short arm of chromosome 3 and the proximal long arm of chromosome 4.

**Figure 3.**
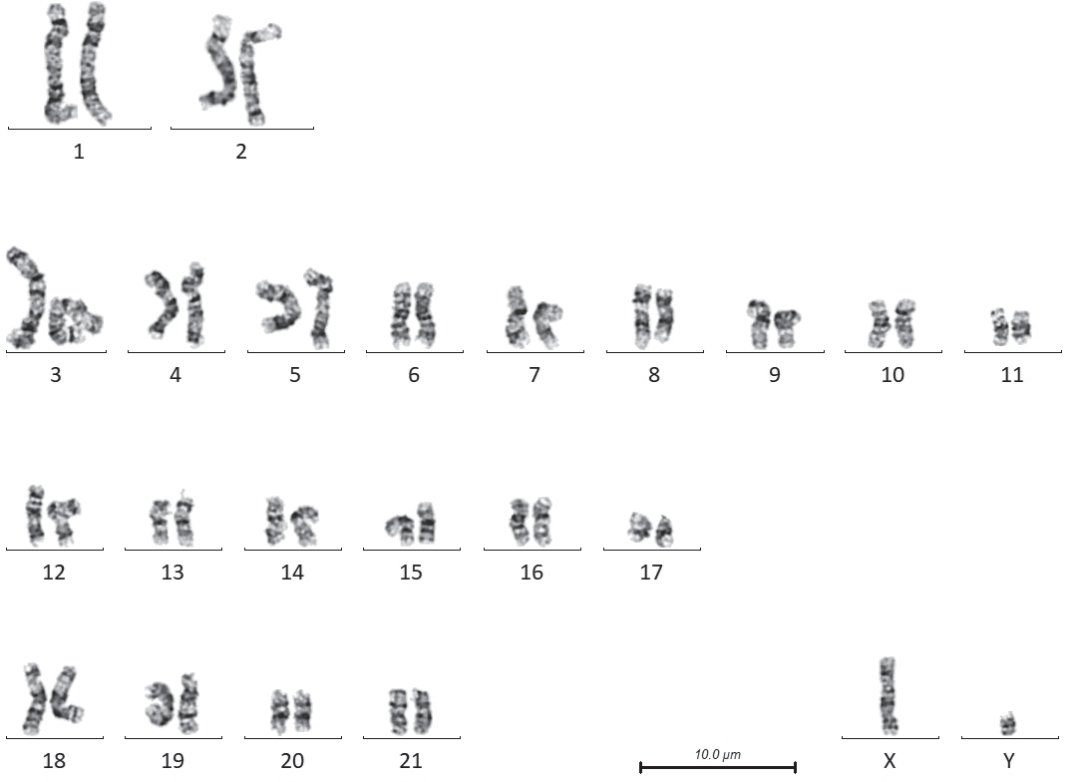
G-banded karyotype of male *T. australis* (BD01).

**Figure 4.**
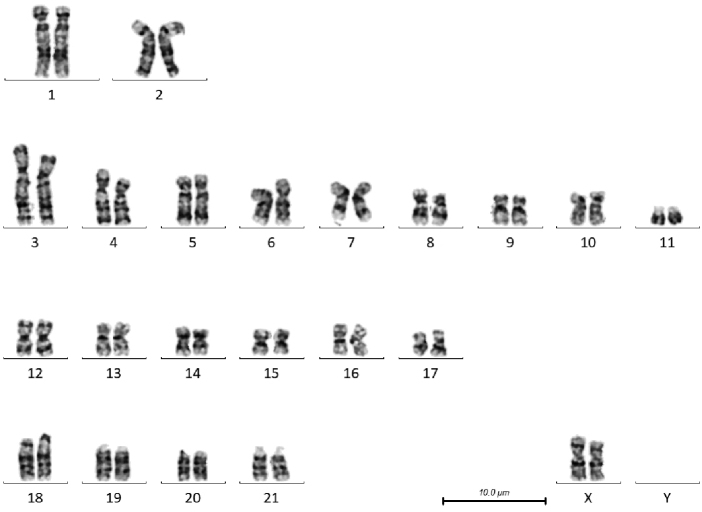
G-banded karyotype of female *T. australis* (BD04). Note the size polymorphism in the distal short arm of chromosome 2, and the short and long arms of chromosome 4.

**Figure 5.**
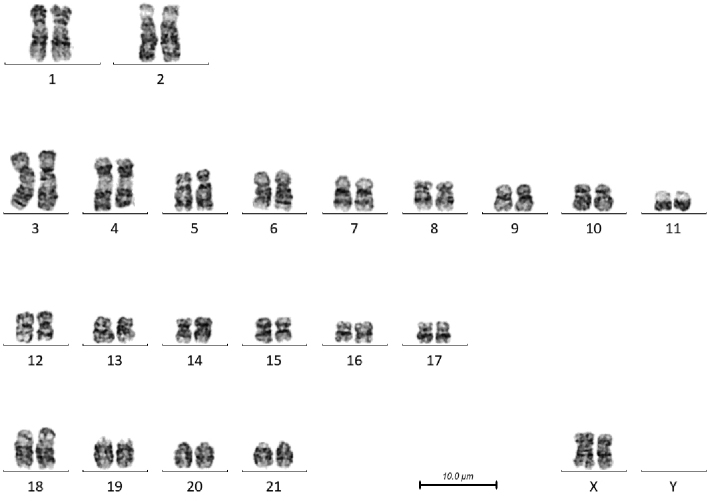
G-banded karyotype of female *G. griseus* (RD01). Note the size polymorphism in the proximal long arm of chromosome 18.

The diploid number of all 3 species is 44. In all individuals studied, the karyotype consists of 2 large subtelocentric pairs (1–2), 9 submetacentric pairs (3–11), 6 smaller metacentric/submetacentric pairs (12–17), 4 acrocentric pairs (18–21), an X chromosome which closely resembles that observed in many mammalian species, and in the 2 males studied, a small Y chromosome. Five to 22 karyotypes per individual were prepared, depending on the availability of suitable metaphases, and these showed a consistent karyotype in each case. A representative karyogram from each of the five individuals studied is presented in Figs 1–5.

There are a number of heterochromatic variants visible in these individuals. In the male *T. truncatus* there is a size polymorphism in the distal short arm of chromosome 6, the chromosome on the right has a larger G-negative band, and in the female, the short arm of chromosome 3 of the chromosome on the right has a larger pale band between the two dark bands, and the proximal long arm of chromosome 4 has a larger G-negative band just below the centromere. In the female *T. australis*, there are variant heterochromatic regions in the distal short arm of chromosome 2, where the chromosome on the right has a larger grey band distally, and the short and long arms of chromosome 4, where the chromosome on the right has a smaller pale band at the end of the short arm, and a smaller pale region just below the centromere. *G. griseus* has a variant on the proximal long arm of chromosome 18, the G-band negative region being larger in the chromosome on the right.

**Figure 6.**
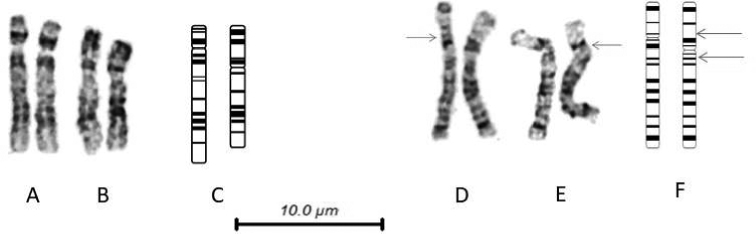
**A** chromosome 1 from **A***T. truncatus***B***G. griseus***C** idiogram of chromosome 1 from *T. truncatus* to the left, *G. griseus* to the right **D** chromosome 2 from *T. truncatus***E***T. australis*, with arrows indicating the position of the centromere **F** idiogram of chromosome 2 from *T. truncatus* to the left, *T. australis* to the right, with arrows indicating possible breakage points of a pericentric inversion.

Apart from the size polymorphisms attributable to heterochromatin variants, the results show that chromosome 1 in *G. griseus* has a significantly different morphology from the two *Tursiops* species. In the *Tursiops* karyograms, the short arm consists essentially of a proximal dark and distal light band, with a pale centromeric region, and a prominent dark band on the proximal long arm. In the *G. griseus* karyogram, the short arm has a darker distal region and a thin dark band in the proximal region, and it is also slightly longer. The centromeric region of *G. griseus* is not as distinctly pale, and there is no proximal dark band on the long arm. The remainder of the long arm is similar, but not completely identical. Overall, the chromosome is slightly shorter in *G. griseus*. Figure 6A–C compares an example of chromosome 1 from *T. truncatus* and *G. griseus*, together with an ideogram showing the differences in banding pattern.

**Figure 7.**
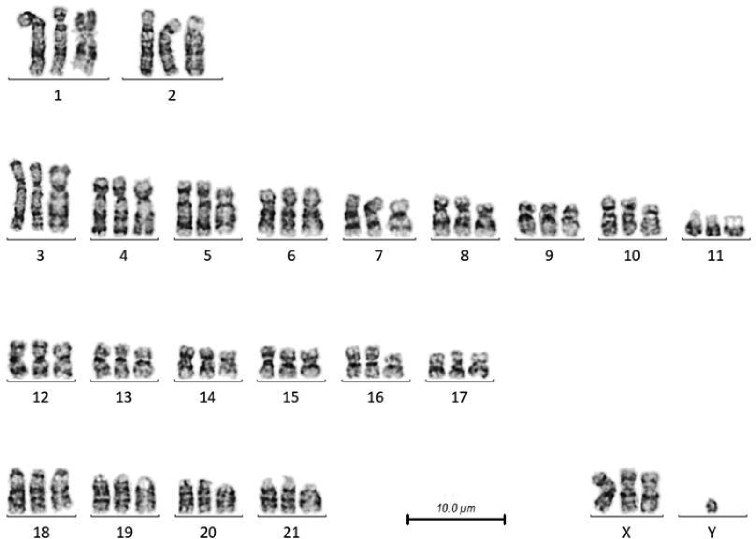
Composite karyogram of the 3 studied species, with 1 homologue of each chromosome presented. Chromosomes from male *T. truncatus* are to the left, female *T. australis* in the middle, and female *G. griseus* to the right.

In both male and female karyograms of *T. australis*, the dark band on the proximal long arm of chromosome 2 in *T. truncatus* and *G. griseus* is present on the proximal short arm. Figure 6D–F compares an example of chromosome 2 from *T. truncatus* and *T. australis*, and an ideogram indicating the banding pattern of the chromosomes. Figure 7 shows a composite karyogram with one homologue from each of the 3 species.

## Discussion

The karyotypes of the three species of dolphin studied here are very similar, all having the same chromosome number (2n = 44) and gross morphology. It is only when studying the detail of the G-banding pattern that differences become apparent. This can be readily visualized by referring to Figure 7, in which the banding pattern of the combined karyograms is apparently identical, with the exception of the chromosomes 1 and 2. The level of banding achieved is, compared to human karyotyping, standard resolution, so greater resolution would allow more precise identification of potential areas of difference. To achieve G-bands, we have used a modification of the trypsin method (Seabright 1971), which produces a banding pattern where GC rich DNA stains pale, and AT rich DNA is dark. The replication method used by Bielec et al. (1997), stains early replicating DNA pale, and late replicating DNA dark, so while the results are broadly consistent, there are differences, for example, heterochromatin can be pale by G-bands, but is dark using replication banding, so this has to be taken into account when comparing karyograms prepared by the two methods.

As the number of individuals available is limited, reasons other than interspecific differences for the observed variation need to be considered. The presence of isolated populations can be a source of intraspecific variation, however in the karyotypes of the individual pairs studied, there was no heteromorphism that could not be assigned to heterochromatic size, relating the variant regions to the C-banded karyogram of *Tursiops gilli* Dall, 1873, now reclassified as *T. truncatus*, depicted in Arnason (1974).

Chromosome 1 appears very similar in *T. truncatus* and *T. australis*, and also, from the literature, in the delphinids *S. clymene*, and *L. albirostris*, and in the harbor porpoise *P. phocoena* (in the latter karyogram the short arm is smaller, lacking the prominent dark band, and the distal C-band positive region is lacking) (Arnason 1980), but is significantly different in the individual of *G. griseus* here analysed. Examination of the karyotypes of apparently related species may assist in determining whether the banding pattern of this chromosome is unique to *G. griseus*, or present in other species, which would indicate an evolutionary relationship.

The proximal dark band on chromosome 2 is on the long arm in *S. clymene*, *L. albirostris*, *P. phocoena* (Arnason 1980) and *T. truncatus* (Bielec et al. 1997), and in *G. griseus* in this study. The pericentric region of this chromosome does not appear to contain a significant C-band positive block, although it has heterochromatic regions in proximity on either side (Arnason 1980), so pericentric inversion of heterochromatin would not explain the different morphology. The simplest explanation is a small pericentric inversion in *T. australis*, however a more complex rearrangement cannot be excluded. Pericentric inversions can occur and be inherited within a species, but are very rarely homozygous in one individual. In this instance, the two animals, although both captive, originated from different locations, and both were homologous for the rearrangement, so a population variant appears unlikely. This finding thus may provide a marker which differentiates *T. australis* and *T. truncatus*. It may also confirm a relationship between *T. australis* and *T. aduncus*, if *T. aduncus* is shown to have the same banding pattern of chromosome 2 as that of *T. australis*. Cytogenetic investigation of *T. aduncus*, together with more individuals of *T. australis*, could therefore clarify the taxonomic position of *T. australis*.

Hybrids between dolphin species occur rarely in the wild, more frequently in captive animals. In captivity, the most frequently observed hybrids result from crosses between *T. truncatus* and *G. griseus* (Espada et al. 2019). Our preliminary observations of banding pattern in these species indicate that hybrids would be recognizable cytogenetically, and the degree of difference in chromosome 1 structure in the two species suggests that meiotic pairing, and thus fertility of a hybrid, would be unlikely.

## Conclusion

The three species of dolphin species described here can be distinguished by their banding pattern, these differences being consistent in all cells within the individuals studied. The small number of individuals analysed makes it premature to draw firm conclusions, but it appears that these differences may potentially have use as an additional tool in determining the species of a particular animal where this is unclear, and in assessment of hybrids. Study of further individuals of these species, and of other dolphins, would enable karyotypic variation to be added to molecular and morphological differences in establishing the evolutionary relationships within this group. In the light of the study by Moura et al. (2020), the morphology of chromosome 2 of *T. aduncus* would be of particular interest in establishing the lineage of *T. australis*.
